# Fundamental mechanism for all-optical helicity-dependent switching of magnetization

**DOI:** 10.1038/srep41294

**Published:** 2017-01-24

**Authors:** Xiang-Jun Chen

**Affiliations:** 1 Department of Physics, Jinan University, Guangzhou 510632, P. R. China

## Abstract

Switching magnetizations with femtosecond circularly polarized lasers may have revolutionary impacts on magnetic data storage and relevant applications. Achievements in ferrimagnetic and ferromagnetic materials of various structures strongly imply a general phenomenon of fundamental atom-laser interaction. Rotating an atom’s wave function with the rotating electric field of a circularly polarized laser, I show the quantum mechanics for the atom is equivalent to that in a static electric field of the same magnitude and a tremendous static magnetic field which interacts with the atom in somewhat different ways. When some conditions are satisfied, transitions of atoms in these two crossed effective fields lead to a highly nonequilibrium state with orbital magnetic moments inclining to the effective magnetic field. The switching finally completes after the pulse duration via relaxation.

The pursuit for a shorter time in switching magnetization by using a shorter magnetic pulse was found to be limited to 2 picosecond^1^. Below this limit, femtosecond circularly polarized laser (CPL) is being demonstrated to be a promising alternative in both ferrimagnetic^2^ and ferromagnetic^3^ materials. The physics for this all-optical helicity-dependent switching (AO-HDS) of magnetization, being practically and fundamentally interesting, has not yet been well understood, largely because in the femtosecond scale some paradigmatic issues in magnetism, such as the spin-orbit interaction, the exchange coupling between atoms, and the macrospin approximation as foundation for applying the Landau-Lifshitz-Gilbert equation, might not be treated conventionally^2^. The fact that the AO-HDS can be achieved in these two types of magnetic orders with completely different exchange couplings signals a fundamental mechanism at the level of atom-laser interaction. The major action of a CPL on an atom is a rotating electric field which becomes static when rotating the atom’s wave function with it, in return for a huge effective magnetic field. I found combined action of them provides a fundamental mechanism for the phenomenon, well explaining important experimental facts such as the highly nonequilibrium state in the pulse duration, the narrow range of laser intensity in which the AO-HDS can occur, and the reason why the final switch occurs long after the pulse. In what follows, I first review important experimental observations to be explained, then deduce the effective Hamiltonian for an atom in the rotating electric field, and finally describe the physics during the switching, suggesting crucial conditions for achieving the AO-HDS.

## Questions arose from experiments

Manipulating magnetization by using a CPL is not a new story. It had been showed early in 1960s that the interaction between a time-varying electromagnetic field and a nondissipative medium can be described by a phenomenological potential, based on classical electrodynamics^4^,^5^,^6^ with a quantum mechanical verification^7^. The Faraday effect (FE), discovered in 1864 by M. Faraday in which a magnetically ordered medium can change the polarization of an incident light, was explained with the potential. The potential, acting as an effective static magnetic field, also predicted the inverse Faraday effect (IFE): an incident CPL can generate a helicity-dependent magnetization in the medium, which was subsequently achieved in paramagnetic solids^8^ and plasmas^9^. The phenomenological parameters in the potential can be measured from the FE, providing an estimation for the induced effective magnetic field in the IFE.

Manipulating magnetically ordered materials with femtosecond lasers began with the first successful demagnetization of a Ni film with a 60 fs laser in 1996^10^. Switching magnetizations by using a femtosecond CPL has been achieved subsequently in ferrimagnetic^2^ and ferromagnetic^3^ materials with various structures, within a narrow range of laser intensity via a highly nonequilibrum process while the actual switching occurs long after the optical pulse is gone^11^. The phenomenon was first analysed with the phenomenological theory for the IFE^4^,^5^,^6^,^7^, in which the effective magnetic field was estimated to be several to 20 T^11^. However, if such a femtosecond switching were achieved by means of a conventional precessional motion, the magnetic field should have been above 10^2^ T and an unrealistically strong damping should have existed in the material^11^. Moreover, a normal magnetic field of this order would have lead to a momentary collapse of the magnetic order, giving rise to no deterministic switching^1^. Laser heating was thus suspected to have been assisting the switching^2^ but this assumption was not supported by subsequent experiments^12^. Now the switching has been basically regarded as an all-optical effect^3^ but a consensus description has not yet been reached despite many theoretical efforts have been devoted to it^13^,^14^,^15^,^16^,^17^,^18^,^19^,^20^,^21^. To describe the AO-HDS, in my opinion, at least the following questions arose from experiments must be answered:

1. Is the AO-HDS caused by a huge effective magnetic field? How can a CPL induce it?
2. What happened in the highly nonequilibrum process in the pulse duration? Why the switching can occur from it after the pulse duration?
3. Why the AO-HDS can only occur in a narrow range of laser intensity?

## Effective fields

**Figure 1:**
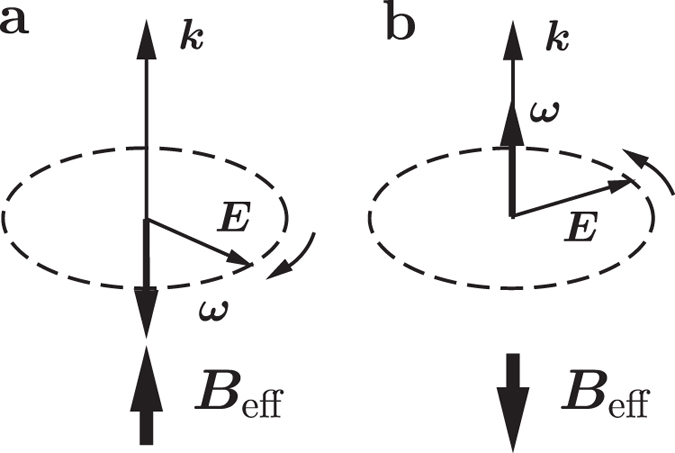
Relation between the helicity of the laser, represented by the angular velocity of the rotating electric field, and the effective magnetic field. (**a**) Left-handed. (**b**) Right-handed.

Exchange couplings in ferrimagnetic materials tend to make magnetic moments of neighbouring atoms oppositely orient while those in ferromanetic materials tend to align magnetic moments of neighbouring atoms. As the AO-HDS can occurs in these two different magnetic orders, we should try to find answers for these questions from fundamental atom-laser interaction in stead of details of materials. A typical CPL used in these experiments is so strong that its major actions on atoms can be treated as a classical rotating electric field,


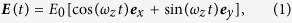


where the *z* axis is chosen on the direction of the wave vector ***k**, **e***_*x*_ and ***e***_*y*_ are unit vectors of the *x* and *y* axises, *E*_0_ is its amplitude, and the helicity of the field can be indicated by its angular velocity ***ω***, as shown in Fig. 1 (the convention for helicity is that typically used in particle physics). Its spatial-dependence is conventionally neglected because the size of an atom is much smaller than the wavelength of the laser. In the classical regime, the rotating field simply drives an electron to move in a circle^22^, generating magnetic dipoles inclining to −***ω*** in a free electron gas, averaged to a helicity-dependent magnetization^23^ identical to that derived from classical electrodynamics based on the Drude model^24^. Quantum correspondence of this simple classical picture is worthwhile considering. To understand the effect of this rotating electric field on spin, we should start from the Dirac equation. In ***E***(*t*) whose corresponding scalar and vector potentials can be chosen as zero and


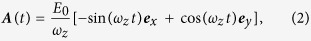


respectively, the Dirac Hamiltonian for an atom with only one electron in its outermost shell reads


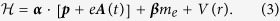


Here I use the natural units, 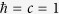. *V*(*r*) is the potential between the electron and the atomic core, assumed to be spherically symmetric. *m*_*e*_ and −*e* stand for mass and charge of the electron respectively, and


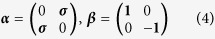


in which **1** stands for a 2 by 2 unit matrix and ***σ*** denotes the Pauli sigma matrix. The Dirac equation with (3) has been exactly solved for the case of *V*(*r*) = 0 25. Here I show the general case of *V*(*r*) ≠ 0 can be transformed to a time-independent one, which is nearly solvable in the non-relativistic limit. Under a unitary transformation on the wave function, *ψ* → *e*^−*i*Θ^*ψ*, the Hamiltonian becomes


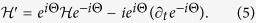


Choosing Θ = *t**ω*** **·** ***J*** is to rotate the wave function with ***ω***, in which ***J*** is the total angular momentum, i.e., sum of the orbital angular momentum ***L*** and the spin angular momentum ***S*** = ***σ***/2. The Hamiltonian is unsurprisingly transformed by this transformation to a time-independent one (see Supplementary Information):


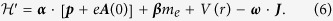


Then, with a gauge transformation on 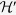, Θ = *e**A***(0) · ***r***, and approximating the Dirac equation to the non-relativistic limit (see, e.g, ref. 26), we have a Schrödinger equation with an effective Hamiltonian (see Supplementary Information),


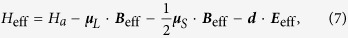


where *H*_*a*_ is the Hamiltonian of the atom including the spin-orbit interaction in the absence of external fields, ***μ***_*L*_ = −*e**L***/(2*m*_*e*_), ***μ***_*S*_ = −*e**S***/*m*_*e*_ and ***d*** = −*e**r*** stand for the orbital magnetic dipole, the spin magnetic dipole, and the electric dipole of the atom respectively. Now the rotating electric field is transformed to a pair of crossed effective fields, an electric field ***E***_eff_ = *E*_0_***e***_*x*_ and a helicity-dependent magnetic field,


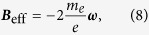


which is obviously the Larmor relation describing the equivalence between a rotating frame and a weak static magnetic field. Typical magnitude of ***B***_eff_, 2.7 × 10^4^ T for an 800 nm laser, is stronger than any magnetic field created by human being^27^. ***E***_eff_ is order of 10^9^ V/m in a typical CPL with a duration of 100 femtosecond and a fluence of 500 mJ/cm^2^ (see Supplementary Information).

Lasers used in AO-HDS experiments are usually off-resonant to the materials. When resonances appear because of the action of these effective fields, we must consider the resonant transitions on influence of the CPL, in addition to the effectively stationary *H*_eff_.

## The highly nonequilibrium process of switching

**Figure 2:**
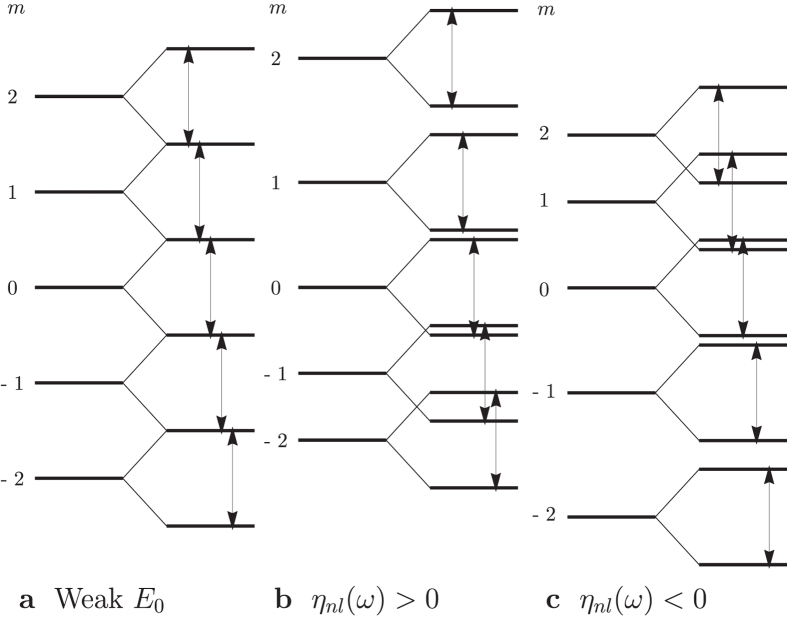
Zeeman levels in the effective magnetic and electric fields of a left-handed laser. Double-headed arrows denote intervals between spin-up and spin-down levels, all equal to *ω*. (**a**) In weak electric field. (**b**) In strong electric field with *η*_*nl*_(*ω*) > 0. (**c**) In strong electric field with *η*_*nl*_(*ω*) < 0.

The ***B***_eff_ is much larger than the field of spin-orbit interaction so that the orbital and spin angular momenta are decoupled. Therefore, energy of the atom without considering ***E***_eff_ is simply the sum of the free atom level *ε*_*nl*_, the orbital Zeeman levels, and the spin Zeeman levels split by ***B***_eff_,


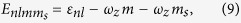


where *n, l, m* and *m*_*s*_ are the usual principal, angular momentum, projection and spin quantum numbers respectively. Magnetic moment of the atom depends on the Zeeman levels it occupied. Zeeman levels for the case of *l* = 2 in a left-handed CPL are showed in Fig. 2(a). They are obviously resonant to the CPL! The electron can resonantly transit among them, giving rise to no macroscopic change in magnetization on the domain radiated by the CPL. This must be guaranteed because ***B***_eff_ is merely the rotating effect of ***E***(*t*), which must cause no observable effect when *E*_0_ is small, no matter how huge it looks. It results from the fact that the spin Zeeman term, the third term on the right-handed side of Eq. (7), is half of that in a normal magnetic field. Another difference of ***B***_eff_’s action from that of a normal magnetic field is the absence of diamagnetic term. Without competition between the diamagnetic term and the paramagnetic term, the random switching induced by a normal strong magnetic pulse^1^ should not appear, I argue.

Therefore, to achieve the AO-HDS, ***E***_eff_, which can be treated as a perturbation comparing to the electric field of 10^11^V/m inside a typical atom, must be able to detune the resonances of Zeeman levels to the CPL. However, it can only perturb the orbital Zeeman levels because of the decoupling between orbital and spin angular momenta. To detune the resonances, the orbital Zeeman levels obviously need to be nonlinearly shifted, that is, in the sense of perturbation theory, the second order corrections to energy eigenvalues must be significant. Considering a hydrogen-like atom as an example, the second order correction 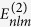 can be routinely obtained by standard technique, in which only the quadratic part of *m*,


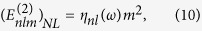


is responsible for the detuning (see Supplementary Information). Here I define a nonlinear shifting factor:


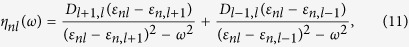


with


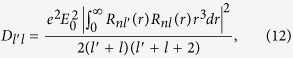


where *R*_*nl*_(*r*) is the radial wave function of the atom. *η*_*nl*_(*ω*) is the crucial parameter for the AO-HDS, depending on the atom’s state, sensitive to the intensity and frequency of the CPL. *η*_*32*_ is roughly estimated to be the order of 10^−1^ eV for a hydrogen-like atom (see Supplementary Information).

Now we can qualitatively understand what had happened in the highly nonequilibrium process of switching. Going on the the case of *l* = 2 as an example, when *η*_*nl*_(*ω*) > 0, intervals between orbital levels of *m* > 0 are widened while those of *m* < 0 are narrowed, leading to level crossings, as shown in Fig. 2(b). Assuming that the atom is initially at state |2, ↑〉, that is, its magnetic moment is opposite to ***B***_eff_, it will initially flipping between |2, ↑〉 and |2, ↓〉, a resonant Rabi oscillation (see e.g. ref. 28), having about 50% chance of occupying |2, ↓〉. If it can be damped from |2, ↓〉 to |1, ↑〉, it will begin flipping between |1, ↑〉 and |1, ↓〉. Similar process leads to flipping between |0, ↑〉 and |0, ↓〉 with no return in orbital levels. However, after that, because of the level crossing, the atom has chances of occupying each levels below |0, ↑〉 because there exist routes such as damped to |−2, ↑〉 from |0, ↓〉, flipping between |−2, ↑〉 and |−2, ↓〉〉, damped to |−1, ↓〉 from |−2, ↑〉, flipping between |−1, ↑〉 and |−1, ↓〉, and then damped back to |0, ↓〉 from |−1, ↑〉. This, in addition to laser heating, can explain the the highly non-equilibrium state on the domain radiated by the CPL^11^. A net magnetization parallel to ***B***_eff_ is generated on this stage, as average of magnetic dipoles due to orbital momenta of atoms distributing in states of *m* ≤ 0. This well explains the magnetizations induced in plasmas and paramagnetic materials and is a very good correspondence of the classical picture^23^.

The key to achieve what described above is to adaptively adjust the nonlinear shifting factor *η*_*nl*_(*ω*) according to energy levels and damping, so as to substantially split the levels of |1, ↓〉 and |0, ↑〉 while keeping the gap between |2, ↓〉 and |1, ↑〉 not too wide for the atom to be damped across in femtosecond scale. This answer the question why the AO-HDS can only occur in a narrow range of laser intensity^2^,^3^. Thanks to the resonant spin flipping between spin-up and spin-down levels, the atom just needs to be damped across the gap between |2, ↓〉 and |1, ↑〉 and the gap between |1, ↓〉 and |0, ↑〉, unrealistically strong damping estimated in ref. 11 is not needed. The situation of *η*_*nl*_(*ω*) < 0, with Zeeman levels shifted as Fig. 2(c), is obviously unfavourable because the atom has a chance to transit among states of *m* ≥ 0.

If the above dynamics can be achieved within the pulse duration of the CPL. After the pulse duration, in ferrimagnetic and ferromagnetic materials, as magnetic moments of atoms have been switched to states inclining to ***B***_eff_, they may relax to the direction of ***B***_eff_ through spin-orbit interactions and electron-electron interactions in each atom, as well as exchange couplings between atoms.

## Discussion

The present effective field approach provides a simple picture of atom-CPL interaction, which qualitatively explains crucial experimental facts of the AO-HDS and may be helpful to understand relevant phenomena of atom-CPL interaction. To quantitatively consider the AO-HDS in real system by using this simple theory based on a simple model of one-electron atom, many details of materials and dynamic processes should be taken into account.

Ignoring exchange couplings between atoms, comparing to ***B***_eff_ of 10^4^ T, may not be a good approximation in some ferromagnetic materials in which the Weiss field, as a measure of the exchange coupling, can reach 10^3^ ~10^4^ T, corresponding to Curie temperature of 10^3^ K^29^. However, ferromagnetic materials used for data storage are those with Curie temperature of 400 ~ 600 K, that is, the Weiss fields are about 10^2^ ~10^3^ T. Exchange coupling is a small effect comparing to ***B***_eff_ but may cause collective switch within the same magnetic domain.

The effective fields are derived on the assumption that *V*(*r*) is spherically symmetric. This condition can be extended to that *V*(*r*) possesses cylindrically symmetry around the wave vector of the CPL, such as the case of anisotropic ferromagnetic materials with easy-axis normal to the surface. For a multi-electron atom, *V*(*r*) could be understood as the pseudo-potential, as in some *Ab Initio* calculation techniques.

To verify the present mechanism for real system, *Ab Initio* calculation for the nonlinear shift factor *η*_*nl*_(*ω*) in various materials, such as those in ref. 30, may be necessary, where effect of spin-orbit interaction and energy band structure should be considered in calculation of *ε*_*nl*_ and the radial wave function. However, *Ab Initio* calculation in a pair of crossed electric and magnetic fields might still be a challenge.

In the analysis of electron transitions, I only consider two major processes: resonant Rabi oscillation and damping. Transitions between non-resonant levels may also occur. The electron may also be excited to upper levels lower than the energy of the photon or even tunnel to upper levers higher than the energy of the photon. These minor processes, with transition rates much less than those of the resonant Rabi oscillations, might not have significant influences on conclusion of the present theory. However, their effects can be studied for specific materials when energy levels are quantitatively known. Details of dampings in materials are also important issues^21^,^30^. The damped transition from |*m*, ↓〉 to |*m*−1, ↑〉 is accompanied by changes in spins and orbital angular momenta. It is challenging to understand what kinds of dampings can yield these changes. Line widths due to damping should also be considered in details of the dynamics.

In conclusion, in the present mechanism, the AO-HDS is achieved in two stages. The first one, in the pulse duration, is dominated by interactions of individual atoms with the CPL, which effectively acts as a static pair of orthogonal electric and magnetic fields. Due to appropriate dampings and resonant spin flipping, atoms in the radiated area are brought to various states with orbital magnetic moments inclining to the effective magnetic field, that is, the switching is more than half done in this stage. The switching completes via relaxation at the second stage after the pulse duration, depending on details of materials.

## Additional Information

**How to cite this article**: Chen, X.-J. Fundamental mechanism for all-optical helicity-dependent switching of magnetization. *Sci. Rep.* **7**, 41294; doi: 10.1038/srep41294 (2017).

**Publisher's note:** Springer Nature remains neutral with regard to jurisdictional claims in published maps and institutional affiliations.

## Supplementary Material

Supplementary Information
