# Supplementary material for: Fundamental mechanism for all-optical helicity-dependent switching of magnetization
Source: Sci Rep. 2017 Jan 24;7:41294. doi: 10.1038/srep41294 (PMC5259757; doi:10.1038/srep41294)
Supplement: Supplementary Information [file srep41294-s1.pdf]

Supplementary Information:

Fundamental mechanism for all-optical helicity-dependent switching of magnetization

Xiang-Jun Chen
Department of Physics, Jinan University, Guangzhou 510632, P. R. China

This supplementary information provides some detailed quantum-mechanical calculations for the main article.

A. The effective Hamiltonean

The Hamiltonian for an atom in the field,

$$\boldsymbol{A}(t) = \frac{E_0}{\omega_z}\left[-\sin(\omega_z t)\boldsymbol{e}_x + \cos(\omega_z t)\boldsymbol{e}_y\right], \tag{1}$$

consists of two parts,

$$\mathscr{H} = \mathscr{H}_0 + \mathscr{H}_1(t), \tag{2}$$

in which

$$\mathscr{H}_0 = \boldsymbol{\alpha}\cdot\boldsymbol{p} + \boldsymbol{\beta} m_e + V(r), \quad \mathscr{H}_1(t) = e\boldsymbol{\alpha}\cdot\boldsymbol{A}(t) = e(\alpha_x A_x + \alpha_y A_y) \tag{3}$$

Transformation on the Hamiltonian can be calculated with the Baker-Hausdorff lemma:

$$e^{i\Theta}\mathscr{H}e^{-i\Theta} = \mathscr{H} + i[\Theta,\mathscr{H}] + \frac{i^2}{2!}[\Theta,[\Theta,\mathscr{H}]] + \cdots + \frac{i^n}{n!}[\Theta,[\Theta,\cdots,[\Theta,\mathscr{H}]\cdots]] + \cdots. \tag{4}$$

For

$$\Theta = \Theta_1 = t\boldsymbol{\omega}\cdot\boldsymbol{J} = t\omega_z L_z + t\omega_z\sigma_z'/2 \tag{5}$$

where

$$\boldsymbol{\sigma}' = \begin{pmatrix}\boldsymbol{\sigma} & 0\\ 0 & \boldsymbol{\sigma}\end{pmatrix}, \tag{6}$$

noting that

$$[J_z,\mathscr{H}_0] = 0, \quad [\sigma_l',\alpha_m] = 2i\varepsilon_{lmn}\alpha_n, \tag{7}$$

where ε_{lmn} is the Levi-Civita symbol,

$$\underbrace{[\sigma_z'/2,\ldots[\sigma_z'/2,}_{\text{odd fold}}\ \alpha_x A_x + \alpha_y A_y]\ldots] = i\alpha_y A_x - i\alpha_x A_y, \tag{8}$$

and

$$\underbrace{[\sigma_z'/2,\ldots[\sigma_z'/2,}_{\text{even fold}}\ \sigma_x A_x + \sigma_y A_y]\ldots] = \alpha_x A_x + \alpha_y A_y, \tag{9}$$

we have

$$e^{i\Theta_1}\mathscr{H}_1(t)e^{-i\Theta_1} = e(\alpha_x A_x+\alpha_y A_y)\cos(\omega_z t)-e(\alpha_y A_x-\alpha_x A_y)\sin(\omega_z t)=e\alpha_y\frac{E_0}{\omega_z} = \mathscr{H}_1(0). \tag{10}$$

The Hamiltonian becomes time-independent:

$$\mathscr{H}'=e^{i\Theta_1}\mathscr{H}e^{-i\Theta_1}-ie^{i\Theta_1}\partial_t e^{-i\Theta_1}=\boldsymbol{\alpha}\cdot[\boldsymbol{p}+e\boldsymbol{A}(0)]+\boldsymbol{\beta}m_e+V(r)-\boldsymbol{\omega}\cdot\boldsymbol{J}. \tag{11}$$

With a further transformation,

$$\Theta=\Theta_2=e\boldsymbol{A}(0)\cdot\boldsymbol{r}=\frac{eE_0}{\omega_z}y, \tag{12}$$

noting,

$$[\Theta_2,\boldsymbol{\alpha}\cdot\boldsymbol{p}]=i\frac{eE_0}{\omega_z}\alpha_y=ie\boldsymbol{\alpha}\cdot\boldsymbol{A}(0),\quad [\Theta_2,L_z]=i\frac{eE_0}{\omega_z}x, \tag{13}$$

we have

$$\mathscr{H}''=e^{i\Theta_2}\mathscr{H}'e^{-i\Theta_2}=\mathscr{H}_0+E_0(ex)-\boldsymbol{\omega}\cdot\boldsymbol{J} \tag{14}$$

In the non-relativistic limit, the top component of the Dirac equation reduces to a Schrödinger equation with an effective Hamiltonian,

$$H_{\text{eff}}=H_a-\boldsymbol{\mu}_L\cdot\boldsymbol{B}_{\text{eff}}-\frac{1}{2}\boldsymbol{\mu}_S\cdot\boldsymbol{B}_{\text{eff}}-\boldsymbol{d}\cdot\boldsymbol{E}_{\text{eff}}, \tag{15}$$

where

$$H_a=\frac{p^2}{2m}+V(r)+\frac{1}{4m_e^2 r}\frac{dV(r)}{dr}\boldsymbol{S}\cdot\boldsymbol{L} \tag{16}$$

is the Hamiltonian of the atom including the spin-orbit interaction in the absence of external fields,

$$\boldsymbol{\mu}_L=-e\boldsymbol{L}/(2m_e),\quad \boldsymbol{\mu}_S=-e\boldsymbol{S}/m_e,\quad \boldsymbol{d}=-e\boldsymbol{r},\quad \boldsymbol{E}_{\text{eff}}=\boldsymbol{E}(0)=E_0\boldsymbol{e}_x.$$

B. The nonlinear shifts

The effective Hamiltonian can be divided into an solvable part and a perturbation part:

$$H_{\text{eff}}=H_0+H' \tag{17}$$

in which

$$H_0=H_a-\boldsymbol{\mu}_L\cdot\boldsymbol{B}_{\text{eff}}-\frac{1}{2}\boldsymbol{\mu}_S\cdot\boldsymbol{B}_{\text{eff}}, \tag{18}$$

$$H'=-\boldsymbol{d}\cdot\boldsymbol{E}_{\text{eff}}=eE_0 r\sin\theta\cos\varphi. \tag{19}$$

Here we drop the spin Zeeman term which does not perturbed by H'. Approximating the atom as an hydrogen-like atom, eigenvalues of H_0 and their corresponding eigenfunctions are:

$$E_{nlm}^{(0)} = \varepsilon_{nl} - m\omega_z, \quad \psi_{nlm}^{(0)} = R_{nl}(r)Y_l^m(\theta,\varphi), \tag{20}$$

in which $R_{nl}(r)$ are the radial wave functions and $Y_l^m(\theta,\varphi)$ are the spherical harmonic functions. By using the formula

$$\sin\theta e^{\pm i\varphi}Y_l^m(\theta,\varphi) = \mp\sqrt{\frac{(l\pm m+1)(l\pm m+2)}{(2l+1)(2l+3)}}Y_{l+1}^{m\pm1} \pm \sqrt{\frac{(l\mp m)(l\mp m-1)}{(2l+1)(2l-1)}}Y_{l-1}^{m\pm1}, \tag{21}$$

one can calculate the matrix elements of H'. The nonlinear parts proportional to m^2 of those nonzero elements are

$$\left(|\langle n,l+1,m\pm1|H'|nlm\rangle|^2\right)_{NL} = \frac{e^2E_0^2\left|\int_0^\infty R_{n,l+1}(r)R_{nl}(r)r^3dr\right|^2}{4(2l+1)(2l+3)}m^2 = \frac{1}{2}D_{l+1,l}m^2, \tag{22}$$

$$\left(|\langle n,l-1,m\pm1|H'|nlm\rangle|^2\right)_{NL} = \frac{e^2E_0^2\left|\int_0^\infty R_{n,l-1}(r)R_{nl}(r)r^3dr\right|^2}{4(2l-1)(2l+1)}m^2 = \frac{1}{2}D_{l-1,l}m^2, \tag{23}$$

where

$$D_{l'l} = \frac{e^2E_0^2|\int_0^\infty R_{nl'}(r)R_{nl}(r)r^3dr|^2}{2(l'+l)(l'+l+2)}. \tag{24}$$

The nonlinear parts of second order corrections to energy eigenvalues[1] are

$$\left(E_{nlm}^{(2)}\right)_{NL} = \sum_{l',m'}{}' \frac{\left(\left|\langle nl'm'|H_p|nlm\rangle\right|^2\right)_{NL}}{E_{nlm}^{(0)} - E_{nl'm'}^{(0)}} = \eta_{nl}(\omega)m^2. \tag{25}$$

Here we define a nonlinear shift factor:

$$\eta_{nl}(\omega) = \frac{D_{l+1,l}(\varepsilon_{nl}-\varepsilon_{n,l+1})}{(\varepsilon_{nl}-\varepsilon_{n,l+1})^2-\omega^2} + \frac{D_{l-1,l}(\varepsilon_{nl}-\varepsilon_{n,l-1})}{(\varepsilon_{nl}-\varepsilon_{n,l-1})^2-\omega^2}. \tag{26}$$

For a $3d$ electron of a hydrogen-like atom,

$$R_{32}(x) = \frac{4}{81\sqrt{30}}\left(\frac{Z}{a_0}\right)^{3/2}x^2e^{-x/3}, \tag{27}$$

$$R_{31}(x) = \frac{4}{81\sqrt{6}}\left(\frac{Z}{a_0}\right)^{3/2}x(6-x)e^{-x/3}, \tag{28}$$

where $x = rZ/a_0$, a_0 is the Bohr radius, and Z stand for the effective atomic number. A direct integration yields,

$$D_{12} = \frac{27e^2E_0^2a_0^2}{8Z^2}. \tag{29}$$

Some numerical estimations

For a laser pulse with a duration of 100 femtosecond and a fluence of 500mJ/cm^2. The intensity I is about

$$I \sim \frac{\text{fluence}}{\text{duration}} \sim 5 \times 10^{12} (\text{W/cm}^2)$$

The corresponding electric field intensity,

$$E_0 = 2745\sqrt{I} \sim 6 \times 10^9 (\text{V/m})$$

Energy of a photon at 800nm is $\omega = 1.55$eV. Choosing $\varepsilon_{32} - \varepsilon_{31} \sim 3$eV and $Z \sim 1$ provides a very rough estimation of $\eta_{32}(\omega)$ as about 10^{-1}eV.

References

1. Schiff L.I. *Quantum Mechanics* Ch.13 (McGraw-Hill, 1968).
